# Epidemiology, Risk Factors, and Biomarkers of Post-Traumatic Epilepsy: A Comprehensive Overview

**DOI:** 10.3390/biomedicines12020410

**Published:** 2024-02-09

**Authors:** Dimitrios Kazis, Symela Chatzikonstantinou, Alin Ciobica, Fatima Zahra Kamal, Vasile Burlui, Gabriela Calin, Ioannis Mavroudis

**Affiliations:** 1 Third Department of Neurology, Aristotle University of Thessaloniki, 541 24 Thessaloniki, Greece; dimitrios.kazis@gmail.com (D.K.);; 2 Department of Biology, Faculty of Biology, Alexandru Ioan Cuza University of Iasi, 20th Carol I Avenue, 700506 Iasi, Romania; alin.ciobica@uaic.ro; 3 Center of Biomedical Research, Romanian Academy, Iasi Branch, Teodor Codrescu 2, 700481 Iasi, Romania; 4 Academy of Romanian Scientists, 3 Ilfov, 050044 Bucharest, Romania; 5 Higher Institute of Nursing Professions and Health Technical (ISPITS), Marrakech 40000, Morocco; 6 Laboratory of Physical Chemistry of Processes and Materials, Faculty of Sciences and Techniques, Hassan First University, Settat 26000, Morocco; 7 Department of Biomaterials, Faculty of Dental Medicine, Apollonia University, 700511 Iasi, Romania; vburlui@gmail.com; 8 Department of Neuroscience, Leeds Teaching Hospitals, Leeds LS2 9JT, UK; 9 Faculty of Medicine, Leeds University, Leeds LS2 9JT, UK

**Keywords:** post-traumatic epilepsy, traumatic brain injury, neuroinflammation, biomarkers in epilepsy, neuroimaging, risk factors for PTE

## Abstract

This paper presents an in-depth exploration of Post-Traumatic Epilepsy (PTE), a complex neurological disorder following traumatic brain injury (TBI), characterized by recurrent, unprovoked seizures. With TBI being a global health concern, understanding PTE is crucial for effective diagnosis, management, and prognosis. This study aims to provide a comprehensive overview of the epidemiology, risk factors, and emerging biomarkers of PTE, thereby informing clinical practice and guiding future research. The epidemiological aspect of the study reveals PTE as a significant contributor to acquired epilepsies, with varying incidence influenced by injury severity, age, and intracranial pathologies. The paper delves into the multifactorial nature of PTE risk factors, encompassing clinical, demographic, and genetic elements. Key insights include the association of injury severity, intracranial hemorrhages, and early seizures with increased PTE risk, and the roles of age, gender, and genetic predispositions. Advancements in neuroimaging, electroencephalography, and molecular biology are presented, highlighting their roles in identifying potential PTE biomarkers. These biomarkers, ranging from radiological signs to electroencephalography EEG patterns and molecular indicators, hold promise for enhancing PTE pathogenesis understanding, early diagnosis, and therapeutic guidance. The paper also discusses the critical roles of astrocytes and microglia in PTE, emphasizing the significance of neuroinflammation in PTE development. The insights from this review suggest potential therapeutic targets in neuroinflammation pathways. In conclusion, this paper synthesizes current knowledge in the field, emphasizing the need for continued research and a multidisciplinary approach to effectively manage PTE. Future research directions include longitudinal studies for a better understanding of TBI and PTE outcomes, and the development of targeted interventions based on individualized risk profiles. This research contributes significantly to the broader understanding of epilepsy and TBI.

## 1. Introduction

Post-traumatic epilepsy (PTE) represents a complex neurological disorder arising as sequelae of traumatic brain injury (TBI), and characterized by recurrent, unprovoked seizures [1,2,3,4]. With TBI being a significant global health concern, the emergence of PTE as a delayed complication poses substantial challenges in terms of diagnosis, management, and prognosis. This paper aims to explore the epidemiology, identify key risk factors, and examine the emerging biomarkers that could potentially aid in the early detection and management of PTE.

Epidemiologically, PTE accounts for a noteworthy fraction of all acquired epilepsies, especially in populations exposed to higher risks of head trauma, such as military personnel and individuals involved in contact sports. The incidence of PTE varies considerably, impacted by elements like the severity of the initial injury, age when the trauma occurs, and the presence of specific intracranial pathologies. The epidemiological trends understanding is crucial in identifying populations at risk and implementing targeted preventive strategies [5,6].

The PTE risk factors are multifaceted, encompassing both clinical and demographic variables. Consistently linked to an elevated risk of developing PTE are elements such as the gravity of the initial brain injury, the existence of intracranial hemorrhages, and the occurrence of early post-traumatic seizures (EPTS). Age, gender, and genetic predispositions also contribute, suggesting complex interactions between environmental influences and inherent susceptibility.

Electroencephalography (EEG), neuroimaging and molecular biology advent have paved the way for the identification of potential biomarkers for PTE. These biomarkers— whether radiological signs indicative of structural brain changes; EEG suggesting neural network dysregulation; or molecular markers reflecting pathophysiological processes such as inflammation, neuronal damage, and aberrant neuroplasticity—enable non-invasive detection of specific patterns in the brain, thereby enriching our understanding of the pathogenesis of PTE but also improving early diagnosis and guiding therapeutic interventions [6,7,8,9,10,11,12].

This study compiles recent knowledge on PTE epidemiology, biomarkers, and risk factors, aiming to provide an in-depth synthesis that illuminates clinical practice and guides future research in this evolving field.

## 2. Epidemiology

Within the realm of mild traumatic brain injury (mTBI), the incidence and prognosis of post-traumatic epilepsy (PTE) present a nuanced clinical picture. Relatively recent research has highlighted that while the overall risk of PTE following mTBI is lower compared to moderate or severe TBI, certain factors significantly elevate this risk. These include the presence and type of intracranial hemorrhage, early post-traumatic seizures (EPTS) within the first week, and a history of alcohol abuse [1,2,3,4].

The nature of seizures post-mTBI varies, with some cases progressing to PTE and others showing no long-term sequelae [5]. Notably, subclinical seizures, identifiable only through EEG monitoring, indicate a more intricate pathophysiology of PTS in mTBI cases [6,7,8].

More than 2% of the population in England and Wales annually visit emergency departments due to head injuries, with a significant proportion being children, and approximately one-fifth of these cases involve a skull fracture or evidence of TBI [9]. Hospital admission is required for about 15% of these cases. Seizures following TBI can vary from immediate concussive episodes to early seizures within the first week and late epileptic seizures occurring more than a week post-injury. Concussive seizures, first documented in Australian football players, are distinct from tonic-clonic seizures and are believed to include primitive reflex elements [10,11]. These seizures typically do not pose a risk for later epilepsy. Among a cohort of 1000 patients with head injuries in Oxford, early seizures were observed in 4.5%, frequently correlated with skull fractures or intracranial hemorrhage [1]. Lee and Lui, [4] observed early seizures in 2.4% of 4232 initially classified mild head injury cases, based on the Glasgow Coma Score (GCS). Subsequent imaging frequently revealed significant hemorrhage, indicating more severe injuries [4]. A substantial study from the National Trauma Data Bank of the USA, involving over 360,000 patients, reported early seizures in only 0.4% of cases [5]. Although this incidence is relatively low compared to other studies, a notable mortality rate of 7% was identified [5]. This study also identified an over-representation of Afro-Caribbean patients and a higher prevalence of alcohol abuse history among those with early seizures.

Investigations into subclinical seizures, detectable through EEG monitoring, have revealed significant findings. For instance, Vespa’s study of patients with moderate to severe TBI showed that 22% experienced seizures, a majority of which were subclinical [6]. Arndt et al. [7] found a similar pattern in children with TBI, where subclinical seizures occurred in 16.1% of those monitored, and any form of epileptic manifestation correlated with poorer TBI outcomes. Continuous EEG monitoring is recommended for patients with moderate to severe TBI where there is a suspicion of seizures [8].

Regarding the broader epilepsy population, previous TBI accounts for approximately 5% of new cases and 20% of prevalent cases [12,13,14]. Young adults and the elderly face a notably elevated risk of developing seizures post-TBI [15]. The highest risk of developing epilepsy is associated with penetrating brain injuries, as demonstrated by the fact that over half of Vietnam veterans with such injuries subsequently developed epilepsy [16]. Studies on TBI patients admitted to trauma units, skewed towards more severe cases, align with larger population-based studies in highlighting the gradations in risk from mild to severe injuries [2,3,15]. Annegers classified TBI into mild, moderate, and severe categories, correlating the severity with the risk of developing seizures, with severe cases showing a significantly higher cumulative risk over 20 years [2].

Yeh et al. [15] in their study, utilizing the ICD-9-50 definition of concussion, further elaborated on risk factors by comparing nearly 20,000 emergency department patients with head injuries to a control group, highlighting the increased risk of epilepsy in cases with more severe forms of brain injury [15]. These findings indicate the need for a nuanced understanding of TBI severity and its relation to the risk of PTE, with considerations for factors like alcohol abuse history and specific types of brain injury.

## 3. Pathophysiology of Post-Traumatic Epilepsy

The role of astrocytes in post-traumatic epileptogenesis (PTE) development has seen a significant paradigm shift, with a growing understanding that extends beyond their traditional role as mere support cells [17]. Recent research underscores their multifaceted functions, particularly in the pathogenic inflammatory response and direct involvement in various neuronal functions, underscoring their integral role in brain activities such as learning, memory, and sleep [18,19,20,21]. Astrocytes, the most prevalent cell type in the brain, are pivotal in maintaining ionic homeostasis, preserving the integrity of the blood-brain barrier, regulating neurotransmitter metabolism, and ensuring neuronal energy supply. Furthermore, they play a critical role in modulating neuronal activity, such as facilitating the exchange of neuronal pyruvate for astrocytic lactate, boosting neuronal metabolism, and participating in synaptic information processing by modulating the uptake and release of neurotransmitters [22,23,24]. They play an essential role in modulating the synaptic availability of neurotransmitters such as glutamate and GABA, thereby influencing synaptic transmission [25,26].

In the context of PTE, the activation of astrocytes is a significant component of the neuroinflammatory response. Gliosis, characteristic of astrocyte activation, is frequently observed after head trauma. Astrocytosis is evident at the site of the primary lesion and in brain regions ipsilateral and contralateral distal to the initial lesion [27,28,29,30,31]. Differentiating seizure-induced gliosis from potentially pro-epileptogenic gliosis, especially in postmortem epileptic brains, poses a challenging task. Yet, analogous gliosis patterns are identified in diverse animal models of TBI, providing opportunities to study potential astrocytic mechanisms in epileptogenesis [32,33,34,35,36,37,38].

In response to TBI, astrocytes react to axonal degeneration, neuronal cell death, and the swift release of inflammatory factors like neurons, cytokines and astrocytes, and chemokines from microglia. This cascade of events can alter the physiological functioning of astrocytes, particularly with respect to signaling and epileptogenesis.

Studies by Steinhauser et al. revealed functional changes in astrocytes in epileptic brains, including reduced potassium currents and altered gap junction coupling, considered key factors in epilepsy development [38,39,40,41]. Activation of astrocytes increases intracellular calcium concentration, leading to increased release of glutamate as a gliotransmitter [42,43]. The release of glutamate may promote neuronal excitotoxicity and increase the potential for seizure generation, potentially involving inflammation-related alterations in receptor expression and disruptions in gap junction coupling [43].

In the context of head trauma, the disconnection of astrocyte gap junctions stands out as a crucial facet of astrocyte activation [44,45].

Furthermore, the role of astrocytic Cx43 hemichannels in seizures has been observed in epileptogenesis models [46]. The use of GAP19, a selective hemichannel inhibitor, demonstrated a reduction in seizure activity by inhibiting these hemichannels [47]. This impact was established to be mediated by the presence of D-serine [47]. Therefore, the neuroinflammatory response triggered by TBI may compromise the capacity of astrocytes to effectively regulate ion balance across the astrocytic syncytium. This is likely associated with issues pertaining to astrocytic gap junctions.

Additionally, responses to neuroinflammation induce both functional and morphological adjustments in astrocytes, with some of these modifications associated with seizures and epilepsy. For instance, in the neuroinflammatory environment, changes in the function and distribution of aquaporin-4 have been suggested as factors contributing to susceptibility to post-traumatic seizures (PTS) [48]. In other models of epilepsy, the absence of aquaporin-4 in mice has been associated with seizure resistance, implying a wider role for astrocytic aquaporins in the regulation of seizures [49].

Another alteration linked to neuroinflammation is astrocyte hypertrophy, which might play a pivotal role in the formation of pro-epileptogenic circuits following head trauma. Research investigations focused on the radial glial-like astrocyte scaffold in the dentate gyrus of the hippocampus after various insults, including head trauma, have shown that these astrocytes hypertrophy and change their orientation [49,50,51,52,53,54,55,56]. The modification in astrocyte structure could serve as an anatomical substrate, offering chemotactic signals that contribute to the abnormal growth of epileptogenic circuits [57,58,59,60].

## 4. The Role of Microglia and Cytokines

Following traumatic brain injury (TBI) or exposure to other pro-epileptogenic triggers, microglial cells undergo rapid activation, a condition that may endure for months or even years post the initial injury [61,62,63,64,65,66]. Microglia, acting as resident macrophages of the central nervous system (CNS), play a crucial role in immune defense. Originating from primitive macrophages of the embryonic yolk sac, they migrate to the developing neuroepithelium and remain in the CNS throughout an individual’s life [67,68]. Besides their immune-related duties, microglia are essential contributors to neuronal proliferation, differentiation, and the establishment of synaptic connections [69,70,71].

Microglial cell activation is a shared characteristic observed in both TBI and the onset of epileptogenesis [72,73]. While not activated, microglia actively examine the cerebral microenvironment through the dynamic extension and retraction of their processes. When activated, they migrate to the site of injury to isolate damaged tissues and phagocytize cellular debris [74,75]. Moreover, they can act as antigen-presenting cells [76]. Studies indicate that inhibiting microglia with minocycline or its derivatives can reduce post-traumatic seizures (PTS), cognitive deficits, and epilepsy [61,77,78,79,80,81,82]. This indicates that microglia directly contribute to inducing neuronal hyperexcitability, although additional research is required for a comprehensive understanding of these mechanisms [81,82,83,84,85].

Microglial activation is typically initiated by chemokines and pro-inflammatory cytokines [86]. This process is coupled with the release of danger-associated molecular patterns (DAMPs) from damaged cells [86]. For instance, DAMP protein high-mobility group box 1 (HMGB1) is released in response to cytokine stimulation by immune cells, neurons, and glial cells [87]. HMGB1 activates Toll-Like Receptor 4 TLR4, and a TLR4 mutation has been associated with seizure resistance in mice [88]. This underscores the importance of microglial signaling in post-traumatic epilepsy (PTE) and highlights the potential to target specific signaling components for therapeutic purposes.

Microglial cells can exert both positive and negative influences on the development of PTE. In vitro studies have established two activation states for microglia: M1 (classical) and M2 (alternative). Both types are present in damaged tissues after TBI [61,89,90].

The M1 profile may confer advantages during the acute response, but the prolonged presence of pro-inflammatory M1 microglia has been associated with persistent neurological dysfunction following injury. Emergence of M2 microglia, in response to certain cytokines, suggests a mechanism for deactivating inflammation and promoting tissue repair [91].

Furthermore, interaction between microglia and astrocytes is crucial in PTE neuroinflammation. Activated microglia can induce the A1 astrocyte phenotype, which is neurotoxic and contributes to neuronal death and synapse disassembly [92].

Cytokines and chemokines play a significant role in PTE. The rapid and sustained release of these inflammatory proteins after TBI is well documented [93,94,95]. Key cytokines such as IL-1β, TGFβ, TNF, IL-6, and IL-10 are consistently elevated after TBI, directly and indirectly increasing neuronal hyperexcitability and contributing to seizures. For instance, IL-1β is raised in TBI patients prone to epilepsy, and it has been shown to decrease seizure threshold in transgenic mice [96,97,98,99,100,101]. Similarly, TGFβ signaling triggers seizures and neuronal hyperexcitability, and its inhibition can reduce the severity of PTS [102,103,104,105]. IL-6, another critical cytokine, is elevated after TBI and associated with an increased susceptibility to seizures [106,107,108,109,110,111,112,113,114,115].

## 5. Post-Traumatic Epilepsy Risk Factors

In their longitudinal study, Khalili et al. [116] investigated the risk factors for post-traumatic epilepsy (PTE) following severe non-penetrating civilian traumatic brain injury (TBI) (Table 1). Conducted at the Hospital of Shahid Rajaee Trauma, associated with Shiraz University of Medical Sciences in Iran, from 2015 to 2019, the research focused on 803 patients with severe TBI, defined by a Glasgow Coma Scale-Motor score of less than six. The primary objective was to identify post-hospitalization seizures as an indicator of PTE. Results revealed that 10.2% (82 patients) of the cohort experienced late post-traumatic seizures (PTS). Significantly, a higher Glasgow Outcome Scale (Extended) score at discharge was inversely associated with the risk of developing PTE (odds ratio [OR] = 0.76, 95% CI: 0.65–0.87; *p* = 0.0001). Trends suggesting associations of PTE with depressed skull fracture (OR = 1.88), epidural hematoma (OR = 1.67), and subdural hematoma (OR = 1.64) were observed but did not reach statistical significance. This conclusion provides valuable insights into the literature on PTE risk following severe non-penetrating TBI. The study’s substantial sample size and the use of logistic regression analysis underscore the complex interaction of factors such as skull fracture and intracranial hematoma in the context of TBI outcomes.

Tingting Yu et al. [119] performed a retrospective clinical analysis to examine the clinical features of PTE and ascertain the factors influencing the latency period of PTE after TBI (Table 1). The investigation involved the analysis of data from individuals with PTE who attended the Department of Epilepsy Outpatient at Beijing Tiantan Hospital from January 2013 to December 2018. The cohort comprised 2862 subjects, predominantly male (78.48%). The average age at the onset of TBI was 21.4 ± 15.1 years, with the highest incidence observed in two age groups: 0 to 12 years and 15 to 27 years. Generalized onset seizures were the most prevalent seizure type among these patients, accounting for 72.82%. Remarkably, 19.95% of PTE patients developed drug-resistant epilepsy. There was notable variability in the latency period for the onset of PTE among subjects, ranging from 8 days to 20 years. The median latency period was 24.0 months, with an interquartile range (IQR) of 5.0 to 84.0 months. Employing Kaplan–Meier curves and Cox proportional hazard regression analysis, the authors identified several factors associated with PTE latency. Gender, age at the time of TBI, occurrence of multiple craniocerebral injuries, TBI severity, acute seizures, types of post-TBI treatments, and the presence of residual disability were all found to be linked to PTE latency. According to the Cox regression model, being aged 18 years or older, undergoing severe TBI necessitating multiple surgical operations, experiencing acute seizures, and having residual disabilities were identified as risk factors associated with a shorter latency period for PTE. This study presents comprehensive insights into the clinical characteristics of PTE, highlighting key demographic trends, seizure types, and risk factors associated with the onset and latency of PTE after TBI. This study contributes significantly to the understanding of PTE, particularly in terms of identifying patients at higher risk for earlier onset post-TBI, thereby informing clinical management and potential intervention strategies [119].

Zaiming Liu et al. [120] conducted a clinical analysis focusing on prognosis and risk factors associated with early Post-Traumatic Epilepsy (EPTE) following TBI. The study analyzed data from 186 patients, identifying crucial risk factors that influence the development of EPTE. Key findings indicated that factors such as the site, type, and degree of injury significantly influenced the likelihood of EPTE. These insights point to the importance of specific injury characteristics in determining the risk of EPTE, highlighting the need for early identification and management of these risk factors to improve patient outcomes and prognosis. This study underscores the significant impact of EPTE on patients with TBI, emphasizing the critical nature of targeted intervention strategies.

Mariajoseph et al. [121] conducted a systematic review and meta-analysis to determine the incidence of PTE in pediatric populations following TBI and to identify potential risk factors associated with PTE in this demographic. The study encompassed an extensive literature search across Web of Science, PubMed, and Embase databases. It specifically targeted randomized cohort studies and controlled trials that evaluated PTE incidence in pediatric TBI patients. The study adhered to PRISMA (Preferred Reporting Items for Systematic Reviews and Meta-Analyses) guidelines, excluding studies with fewer than 10 patients and those without a clearly discernible pediatric cohort. According to the review’s findings, the overall incidence of PTE following pediatric TBI stood at 10%, and the 95% confidence interval (CI) covered a range from 5.9% to 15%. A subgroup analysis of a limited number of studies identified factors that increased the risk of PTE in pediatric patients. Specifically, the occurrence of early seizures was significantly associated with an increased risk of PTE, with a cumulative incidence ratio (CIR) of 7.28 (95% CI = 1.09–48.4, *p* = 0.040). Additionally, severe TBI was linked to a higher incidence of PTE, with a CIR of 1.81 (95% CI = 1.23–2.67, *p* < 0.001). Intracranial hemorrhage was another factor that raised the risk of PTE, with a CIR of 1.60 (95% CI = 1.06–2.40, *p* = 0.024). Additional factors, including neurosurgical intervention and male sex, exhibited a nonsignificant association with an increased incidence of PTE. PTE stands out as a significant chronic complication after childhood TBI, as underscored by Mariajoseph et al. [121], reflecting similar patterns seen in the adult population. The findings emphasize the necessity for additional standardized research on clinical risk factors and the formulation of management guidelines for PTE in pediatric patients who have experienced TBI [121].

Laing et al. [122] conducted a comprehensive study to assess the risk factors, associated mortality, morbidity, and the impact of early posttraumatic seizures (EPTS) to the emergence of PTE in patients with moderate to severe TBI (Table 1). The research utilized data from a cohort study based on an Australian registry, comprising adults aged 18 years and older who had experienced moderate to severe TBI, spanning from January 2005 to December 2019. In the state of Victoria, where the population is 6.5 million, the trauma registry identified 15,152 patients with moderate to severe TBI based on the Abbreviated Injury Scale (AIS) head severity score. EPS in this study were identified using the Tenth Revision, International Statistical Classification of Diseases, and Australian Modification (International Classification of Diseases ICD-10-AM) codes recorded after acute admission. In addition to examining in-hospital metrics, the study delved into 2-year outcomes, particularly focusing on the occurrence of PTE and post-discharge mortality. The researchers employed a selection operator Least Absolute Shrinkage and Selection Operator (LASSO) regression and an adaptive least absolute shrinkage to construct a prediction model for the risk factors of EPS. Out of the 15,152 participants, 416 (2.7%) were identified with EPS, including 27 (0.2%) with status epilepticus. The multivariable analysis indicated significant risk factors for developing EPS, including a higher Charlson Comorbidity Index, subdural hemorrhage, younger age, subarachnoid hemorrhage, TBI resulting from a low fall, elevated Injury Severity Score, along with heightened severity of head injury assessed through the AIS and Glasgow Coma Score. After adjusting for confounders, EPS were linked to elevated rates of ICU admissions and length of stay, ventilation duration, longer hospital stays, and a higher likelihood of discharge to inpatient rehabilitation rather than home. However, there was no significant difference in in-hospital mortality. The 24-month outcomes for TBI admission survivors showed that cases with EPS had poorer results, including higher mortality rates (relative risk [RR] = 2.14; 95% CI, 1.32–3.46; *p* = 0.002), an increased likelihood of developing PTE (RR = 2.91; 95% CI, 2.22–3.81; *p* < 0.001), and a higher ASMs use (RR = 2.44; 95% CI, 1.98–3.02; *p* < 0.001). The prediction model for EPS demonstrated an area under the receiver operating characteristic curve of 0.72 (95% CI, 0.66–0.79), with a sensitivity of 66% and specificity of 73% in the validation set. In summary, Laing et al.’s study delineates crucial risk factors associated with EPS following moderate to severe TBI and establishes a significant connection between EPS and prolonged stays in the ICU and hospital, heightened ICU ventilation, and adverse outcomes spanning 24 months [122]. These results encompass increased mortality rates and the development of PTE. Valuable insights are garnered from this study regarding the implications of EPS in the context of TBI and underscores the need for targeted interventions to improve patient outcomes [122].

Pease et al. [124] conducted a study focused on severe TBI patients, exploring the incidence and associated risk factors of PTS. Encompassing 598 patients with severe TBI, of which 192 died before discharge, the study observed 392 patients, including 18 with early PTS and 98 with late PTS, of which 72 cases involved recurrent seizures. Over a median follow-up period of 3.5 years and 11 encounters, excluding initial inpatient rehabilitation stays, the study revealed a 5% rate of early PTS. Among TBI survivors, the late PTS incidence reached 25% at five years and 32% at fifteen years, with a high recurrence rate of 61% within two years and 82% within ten years. Patients with early PTS had a significantly higher 44% risk of experiencing late PTS within two years compared to those without early PTS. Most patients began taking antiseizure medications (ASMs) after the first late PTS, except in cases of delayed medical care or specific circumstances such as cocaine use or shunt malfunction. Among patients with recurrent seizures, 25 were not on ASM at the time of recurrence for reasons such as never starting, medication shortages, or being weaned by healthcare providers. At the last follow-up, 23 of the 98 PTS patients were receiving multiple ASMs. In their multivariate models, Pease et al. identified age, decompressive hemicraniectomy (DHC), and CNS infection as factors increasing the risk of PTS. Shunt dependence and female gender were linked with a decreased risk of recurrent PTS [124]. This research provides valuable insights into PTS patterns and risk factors after severe TBI, significantly contributing to the understanding of PTE and informing clinical management strategies for these patients [124].

Tao Xu et al. [125] conducted a comprehensive study to understand the risk factors associated with PTE subsequent TBI. Their research encompassed a range of cohort, case-control, and cross-sectional studies, offering a broad perspective on the factors influencing the likelihood of developing PTE. One of the significant findings from their analysis was the higher risk of PTE in men compared to women, with men having a 32% increased risk (relative risk [RR] of 1.32). This gender disparity points to the need for gender-specific approaches in managing and preventing PTE. Additionally, a history of alcohol abuse emerged as a notable risk factor, more than doubling the likelihood of PTE (RR of 2.18), highlighting the impact of lifestyle factors on the development of neurological complications post-TBI. Another critical aspect identified was the role of posttraumatic amnesia, revealing its association with a 31% heightened risk of PTE. The presence of focal neurologic signs post-TBI also increased the risk by 42%. Furthermore, the experience of loss of consciousness at the time of the initial injury was linked to a 62% increased risk of developing PTE. These findings underscore the importance of immediate clinical assessments and interventions following TBI. The study also shed light on the significance of abnormal neuroimaging findings in predicting the risk of PTE. Conditions such as skull fractures, midline shifts, brain contusions, subdural hemorrhages, and intracranial hemorrhages were all identified as strong predictors, with RRs ranging from 1.46 to 2.65. This underscores the crucial role of neuroimaging in the early detection and individuals’ management of risk for PTE. Importantly, Tao Xu et al. [125] found that the risk of PTE remains highest within the first year after TBI but continues to be significantly elevated for more than a decade post-injury. This long-term risk factor is especially pronounced in cases involving skull fractures, mild, and severe brain injuries, suggesting the need for prolonged monitoring and follow-up in these patients. Overall, the meta-analysis by Tao Xu et al. [125] provides valuable insights into the diverse risk factors for PTE, ranging from demographic variables to clinical presentations and neuroimaging findings. These insights are instrumental in forming targeted prevention strategies and treatment approaches for PTE, especially for identifying and managing high-risk populations [125].

## 6. Risk of Epilepsy after Traumatic Brain Injury

Karlander et al. [126] conducted an extensive register-based cohort study to characterize the epilepsy risk in adults following hospitalization for traumatic brain injury (TBI). The research involved the inclusion of all individuals aged 18 to 100 who underwent their initial hospitalization for TBI within Sweden’s national patient register from 2000 to 2010, constituting a comprehensive cohort of 111,947 patients. Each patient was matched with three controls, resulting in a comprehensive set of 325,881 matched controls based on age and sex. The severity of TBI was categorized, and the risk of epilepsy was estimated using Kaplan–Meier curves. Hazard estimation in both univariate and multivariate analyses was conducted using Cox regression. Haut du formulaire.

The findings demonstrated a significant risk of epilepsy post-TBI, with variations based on injury severity. The risk of epilepsy over a 10-year period was 12.9% in cases of focal cerebral injuries, 8.1% in instances of diffuse cerebral injuries, 7.3% for extra cerebral injuries, 2.8% for skull fractures, and 2.6% for mild TBI. In comparison, the corresponding 10-year risk for the control group was only 0.9%. The hazard ratio (HR) for developing epilepsy increased with TBI severity, ranging from 3.0 for mild injury to 16.0 for focal cerebral injury. Additional risk factors were identified in the multivariable analyses, specifically in the realm of central nervous system (CNS) comorbidities. However, even after adjusting for these comorbidities, TBI remained a significant factor. Other risk factors included male sex, older age, the need for mechanical ventilation, and the occurrence of seizures during the index hospitalization. This study provides valuable insights into the elevated risk of epilepsy post-TBI and the impact of injury severity and associated factors on this risk [126].

Annegers and Coan conducted a study aimed at understanding the incidence of TBI and pinpointing the features of brain injuries linked to the development of seizures [127]. Covering the span from 1935 to 1984, the study investigated 5984 instances of TBI documented in Olmsted County, Minnesota. Out of these, 4541 incidents underwent follow-up assessments to monitor the occurrence of seizures. TBIs were categorized by severity: moderate (loss of consciousness from 30 min to 1 day or a skull fracture), mild (loss of consciousness or amnesia lasting less than 30 min), or severe (loss of consciousness for more than 1 day, subdural hematoma, or brain contusion).

Between 1975 and 1984, the peak incidence of TBI reached 800 cases per 100,000 in males aged 15–24. The study found that the relative risk of seizures varied with the severity of the injury. Mild injuries exhibited a relative risk of 1.5, with no significant rise in risk noted after 5 years. The risk for moderate injuries was 2.9, and it was considerably higher at 17.2 for severe injuries. Key risk factors for seizures identified in this study included consciousness loss, amnesia of 1 day or more, skull fracture, or brain contusion with subdural hematoma, and being over 65 years old [127].

Annegers and Coan’s findings underline the significance of TBI as a notable contributor to both the public health landscape and the incidence of epilepsy and seizures. This study provides critical insights into the various risk factors linked to seizure development following different severities of TBI [127].

## 7. Biomarkers

### 7.1. NeuroImaging Biomarkers

In their investigation, Gupta et al. [123] utilized diffusion tensor imaging (DTI) to assess and measure brain abnormalities in individuals with chronic traumatic brain injury (TBI). The study specifically concentrated on participants with and without late posttraumatic epilepsy (PTE) [123]. The study included 23 chronic TBI patients (14 with epilepsy and nine without) along with 11 age-matched controls. The primary objective was to identify microstructural changes beyond those visible on conventional T2 and fluid-attenuation inversion recovery (FLAIR) MRI scans. The researchers calculated ratios of fractional anisotropy (FA) and mean diffusivity (MD) between regions of interest, extending beyond the T2/FLAIR visualized abnormalities, and their corresponding contralateral normal-appearing regions (Table 1). These ratios were then compared among the TBI groups and the control group. Additionally, the volume of tissue exhibiting abnormalities on DTI was measured in these patients. The results revealed significant differences: TBI patients exhibited a lower mean regional FA ratio and a higher MD value compared to controls. Among the TBI groups, those with epilepsy showed a notably lower FA ratio and a slightly higher, though not statistically significant, MD ratio compared to those without epilepsy. Furthermore, the volume of tissue with a low FA value was greater in TBI patients with epilepsy. In conclusion, Gupta et al. found that the severity of injury, as indicated by the increased volume of microstructural damage detected by DTI, is associated with the development of delayed PTE in chronic TBI patients [123]. These results suggest the potential utility of DTI as a valuable tool in predicting epileptogenesis in patients with chronic TBI, providing crucial insights for your paper on mTBI and PTE.

In the realm of TBI, imaging has traditionally focused on the initial formation of primary lesions and the evolution of gliosis. Despite this, there is potential in imaging techniques for monitoring the progression of pathologies linked to PTE. The appeal of neuroimaging biomarkers lies in their noninvasive nature and routine applicability in patient care, enabling the identification of patterns within specific brain structures or across the entire brain. Over the years, CT scans have played a crucial role in evaluating global structural damage following TBI (Table 2). Early CT scans conducted in the early stages have correlated specific findings, including depressed skull fractures, dural penetration, and various types of hemorrhages, with an elevated risk of PTE (Table 2). Even in instances of cortical/subcortical contusions or large temporal lobe lesions, higher rates of PTE have been observed, regardless of the severity of the initial injury [128].

PET imaging, particularly utilizing 18F-Fluorodeoxyglucose (FDG), has proven valuable in exploring inflammatory responses and metabolic consequences following TBI. Patterns of hypometabolism and hyperglycolysis post-TBI have been associated with inflammatory cell proliferation around the impact site [129].

MRI, with a focus on fluid-attenuated inversion recovery techniques and T2-weighted, has demonstrated effectiveness in visualizing gliosis and inflammation post-TBI. Inflammatory cascades triggering the activation of astrocytes and microglia are connected to diverse epileptogenic factors, such as excitotoxicity and mitochondrial dysfunction [130]. Magnetic resonance spectroscopy provides quantitative insights into altered metabolic profiles related to inflammation and hyperexcitability, with documented post-TBI changes in key neurotransmitters and metabolic indicators [131,132].

Structural MRI studies in rats post-TBI have detected minor yet significant hippocampal changes correlating with the incidence of PTE. Quantitative diffusion MRI, particularly with mossy fiber sprouting scores, holds promise in predicting an increased seizure risk after TBI [133].

Functional MRI has been employed to investigate brain connectivity, plasticity, and remodeling post-injury. While studies linking functional changes to epileptogenesis and PTE are limited, alterations in connectivity have been associated with behavioral, cognitive, and motor control impairments [134,135]. Blood-brain barrier (BBB) dysfunction, visualized using fluid-attenuated inversion recovery and dynamic contrast-enhanced MRI techniques, emerges as a critical factor in seizure susceptibility post-TBI. Disrupted BBB function is more prevalent in post-traumatic seizures (PTS) patients compared to those without epilepsy after TBI [136,137].

### 7.2. Electrophysiological Biomarkers

Electrographic biomarkers are increasingly recognized as potential predictors for the initiation of epileptogenesis and seizures, paving the way for the advancement of focused preventive treatments. While currently lacking validated electrophysiological biomarkers for PTE, experimental EEG studies have identified several promising candidates. These encompass alterations in theta oscillations, pathologic high-frequency oscillations (HFOs), modifications in sleep spindle duration, and epileptiform spiking or discharges occurring before the onset of seizures (Table 2).

HFOs, categorized into ripples (80–250 Hz) and fast ripples (250–500 Hz), play a role in both physiological and pathological brain processes. Distinguishing between physiological and pathological HFOs based solely on spectral frequency remains challenging, but an increase in the power of HFOs and rhythmic patterns has been linked to epileptic foci. Various methods, including standard scalp EEG, have demonstrated the long-term detectability of HFOs, suggesting their potential as noninvasive biomarkers for epilepsy [138,139].

Sleep spindles, occurring at frequencies of 10–20 Hz, are generated through interactions between inhibitory neurons in thalamic regions. Notably, spindle frequency increases just before REM sleep. Disruptions in normal spindle activity following TBI could contribute to epileptogenesis, as evidenced by a study that found altered sleep spindle characteristics during the transition from slow-wave N3 to REM sleep in epileptic rats [140,141].

In animal models post-brain injury, the presence of EEG spikes, referred to as interictal spiking, has been noted. These spikes, representing abnormal brain wave fluctuations, are yet to be definitively linked to the onset of spontaneous recurrent seizures (SRS). Additionally, stimulation-evoked hyperexcitability in hippocampal and neocortical regions post-injury has been documented, although the direct association with injury state or epileptogenesis remains unclear [142,143].

Despite these encouraging results, additional experimental studies employing controlled EEG analytics are necessary to identify reliable PTE biomarkers in clinical settings. Machine learning approaches may also aid in classifying these abnormal EEG patterns more effectively [144]. The analysis algorithms, detection protocols standardization, and EEG recordings sampling will be crucial in advancing the discovery of neurophysiologic biomarkers for PTE.

### 7.3. Molecular Biomarkers

Exploring molecular biomarkers offers insights into the severity of TBI, monitoring disease progression, and predicting clinical outcomes. These biomarkers, detectable in various biofluids like blood, cerebrospinal fluid (CSF), plasma, and saliva, encompass a range of molecular entities, comprising extracellular vesicles, microRNAs (miRNAs), cytokines, and proteins (Table 2).

Severe TBIs, characterized by extensive tissue damage, bleeding, and inflammation, significantly increase the risk of PTE. Molecular aftermath of such injuries is reflected in elevated regulators, inflammatory chemo- and cytokines, and bone morphogenic protein levels, aiding in categorizing the injury’s severity and predicting seizure development post-TBI. Critical risk factors for seizure onset include the presence of bone fragments or foreign bodies in brain tissue. Additionally, markers like occludin, claudin-5, VEGF, von Willebrand factor, and aquaporin-4 in serum or CSF may indicate a compromised BBB or vascular injury [145,146,147].

Iron accumulation in the bloodstream, resulting from excessive bleeding, also heightens the risk of PTE due to its cytotoxic effects, leading to mitochondrial dysfunction and oxidative stress. TBI patients with low levels of ceruloplasmin, a protein crucial in iron metabolism, have been associated with increased intracranial pressure and subsequent seizures. Furthermore, neuroinflammation at the impact site correlates with elevated intracranial pressure, and cytokines like fibrinogen, IL-1, CD53, IL-6, TNFα, and MIP1α play significant roles in sustaining this inflammatory response [148,149,150].

miRNAs have emerged as promising molecular biomarkers in neurological disorders due to their stability and presence in biofluids. Studies have identified specific miRNAs associated with epilepsy and status epilepticus conditions, suggesting their potential role in epilepsy pathogenesis. Variations in miR-19-3p and miR-21-5p have been noted in epilepsy conditions, and miR-328-3p proportion bound to argonaute2 protein increases following a spontaneous seizure. Notably, certain miRNAs, including miR-21-5p, miR-27b-3p, miR-93, miR-135a, miR-146a, miR-155, miR-203, and miR-451, are upregulated in both epilepsy and post-brain injury scenarios, suggesting a shared pathological process between these conditions. In contrast, miR-27a-3p, miR-128, and miR-221-3p typically exhibit a decrease in expression. However, the field is still in its early stages, and there is a need for more comprehensive studies to validate these biomarkers for clinical use in PTE [151,152,153].

A systematic review by Vasilieva et al. [153] explored the predictive capability of miRNAs in identifying the risk of epileptogenesis, a phase following brain tissue damage due to TBI, stroke, or infectious diseases, leading to neural network restructuring and epileptiform activity. The review focused on aberrant expression of circulating miRNAs in conditions like epilepsy, TBI, and ischemic stroke, highlighting miR-21, miR-181a, and miR-155. While these miRNAs show promise, the study emphasizes the complexity of using miRNA-based biomarkers for epileptogenesis, considering factors such as sample collection timing, the type of biological fluid used, and other nuances are crucial for accurate interpretation.

In conclusion, although miRNAs such as miR-21, miR-181a, and miR-155 show promise as potential biomarkers for predicting epileptogenesis, their application is still in the early stages. Further detailed and extended research is necessary to fully understand their role and reliability in the context of epileptogenesis.

## 8. Discussion

This paper has synthesized current knowledge on the epidemiology, risk factors, and biomarkers of Post-Traumatic Epilepsy (PTE), providing a comprehensive overview that informs clinical practice and guides future research in this evolving field. The incidence of PTE varies, influenced by factors like the severity of the initial injury, age at the time of trauma, and intracranial pathologies. This variability emphasizes the need for personalized approaches in both preventive and therapeutic strategies.

The multifaceted nature of risk factors for PTE highlights the complexity of the disorder. Clinical and demographic variables like the severity of initial brain injury, intracranial hemorrhages, and early post-traumatic seizures (PTS) have been consistently associated with an increased risk of PTE. Age, gender, and genetic predispositions also play contributory roles, suggesting complex interactions between environmental influences and inherent susceptibility.

Advancements in neuroimaging, EEG, and molecular biology have facilitated the identification of potential biomarkers for PTE, spanning from radiological signs of structural brain changes to EEG patterns indicating neural network dysregulation and molecular markers of inflammation and neuronal damage. The development of these biomarkers is promising for enhancing our understanding of PTE pathogenesis, improving early diagnosis, and guiding therapeutic interventions.

The role of astrocytes and microglia in PTE, highlighted in the paper, underscores the significance of neuroinflammation and its potential as a therapeutic target. The findings presented suggest that targeting specific components of astrocyte and microglia activation could be a viable strategy for preventing or mitigating PTE.

The studies reviewed also shed light on the nuances of PTE risk factors, including the impact of early seizures, the role of specific types of brain injuries, and demographic factors like age and gender. These insights are crucial for developing more effective prevention and intervention strategies.

## 9. Conclusions

In conclusion, Post-Traumatic Epilepsy (PTE) emerges as a complex neurological disorder with multifaceted risk factors and diverse potential biomarkers. This paper has contributed to the understanding of PTE by providing a detailed overview of its epidemiology, risk factors, and emerging biomarkers. The findings underscore the importance of continued research in this field, particularly in refining the identification of at-risk populations, developing predictive biomarkers, and exploring new therapeutic targets. The complexity of PTE necessitates a multidisciplinary approach, combining insights from clinical practice, imaging, molecular biology, and neurophysiology. Future studies should focus on longitudinal analyses to gain a deeper understanding of the long-term outcomes associated with TBI and PTE. Additionally, there is a need for research dedicated to the development of targeted interventions tailored to individual risk profiles. This research not only aids in managing PTE but also contributes to the broader understanding of epilepsy and traumatic brain injuries.

## Figures and Tables

**Table 1:** Key Risk Factors for Post-Traumatic Epilepsy.

Study (Author)	Key Risk Factors	References
Khalili et al.	Higher Glasgow outcome scale at discharge, depressed skull fracture, epidural hematoma, subdural hematoma	[116]
Manninen et al.	Acute and subacute neuropathologic changes, T2 MRI data	[117]
Missault et al.	Subacute neuroinflammation, microstructural changes	[118]
Yu et al.	Male gender, age at TBI, seizure type, latency	[119]
Liu et al.	Injury site, injury type, injury degree	[120]
Mariajoseph et al.	Occurrence of early seizures, severe TBI, intracranial hemorrhage	[121]
Laing et al.	Subdural hemorrhage, younger age, TBI from low fall, higher Charlson Comorbidity Index, subarachnoid hemorrhage	[122]
Gupta et al.	Abnormality beyond T2/FLAIR lesions, decreased fractional anisotropy (FA) ratio, increased mean diffusivity (MD) ratio	[123]

**Table 2:** Comprehensive Overview of Biomarkers and Diagnostic Techniques for Assessing and Predicting Post-Traumatic Epilepsy (PTE).

Category	Biomarkers and Diagnostic Techniques
**Imaging**	- **CT Scans:** Assess global structural damage; identify risk factors like intraparenchymal, depressed skull fracture, dural penetration, subdural, or epidural hemorrhage. - **Positron Emission Tomography, PET Scans:** Visualize inflammatory responses and metabolic impact post-TBI using tracers like 18F-Fluorodeoxyglucose (FDG); reveal hypometabolism or hyperglycolysis. - **Magnetic Resonance Imaging MRI:** Highlight gliosis and inflammation; detect subtle changes in brain structures post-TBI. - **Dynamic Contrast-Enhanced MRI:** Visualize BBB dysfunction; invaluable for assessing disruptions in BBB integrity. - **Diffusion MRI:** Identify alterations in specific brain regions, suggesting seizure risk. - **Functional MRI:** Analyze connectivity and remodeling post-injury; unveil functional changes in the brain post-TBI.
**EEG**	- **High-Frequency Oscillations (HFOs):** Ripples (80–250 Hz) and fast ripples (250–500 Hz) associated with epileptic foci. - **Sleep Spindle Disturbances:** Alterations in sleep spindle frequency and duration linked to REM sleep and epileptogenesis. - **Interictal Spiking:** EEG spikes indicating abnormal brain wave fluctuations. - **Stimulation-Evoked Hyperexcitability:** Demonstrated in hippocampus post-TBI. - **Pathological EEG Patterns:** Including increased HFOs and epileptiform discharges preceding seizure onset.
**Molecular**	- **Cytokines:** IL-1, IL-6, TNFá, etc., indicating inflammation post-TBI. - **Claudin-5, Vascular Endothelial Growth Factor VEGF, Occludin, Aquaporin-4:** Indicators of BBB breakdown and vascular injury. - **Iron Levels:** Reflecting bleeding and risk of PTE. - **miRNAs:** Including miR-21-5p, miR-27b-3p, miR-93, etc., observed in TBI and epilepsy. - **GFAP:** Indicating astrocytic activity, inflammation, and glucose metabolism. - **Extracellular Vesicles:** Carrying DNA, mRNA, miRNA, and other biomolecules. - **tRNA Fragments:** Such as 5′GluCTC, 5′AlaTGC, and 5′GlyGCC.
